# Atlanta Society of Medicine

**Published:** 1886-12

**Authors:** 


### ATLANTA SOCIETY OF MEDICINE.

Officially reported for THE ATLANTA MEDICAL AND SURGICAL JOURNAL.

STATED MEETING, October 5, 1886.

Dr. N. O. Harris, President, in the chair.

Dr. Crow read a paper on

PUERPERAL ECLAMPSIA.

He defined puerperal eclampsia as a convulsion of epileptiform type, characterized by loss of consciousness and of sensibility, together with tonic and clonic spasms, occurring during the puerperal state.

Etiology: This he considers still an unsettled question. He reviewed the different the ories advanced since Rayere, in 1840, and Leur, in 1842, observed that the convulsions were associated with albuminuria; among them were Frerchs, Hulbertsman, Traube, Rosenstein, Jas. Y. Simpson, Meadows, Tyler Smith, Rob't Barnes and Fordyce Barker. All of these, while agreeing in some points, differ in others; so, after reviewing the pathological changes as found on *post mortem* examinations and observing two clinical facts apparently conflicting, *i. e.*, convulsions may occur, with either albuminuria or œdema, while on the other hand albuminuria, even with œdema, often occurs without convulsions. So he advocates the following as the most plausible theory to his mind:

"Several conditions concur in this disorder, which are: (1) the hydræmic state of gestation leading to imperfect nutrition of the nervous centers, increasing (2) the *normal nervous tension* and irritability and (3) the normal vascular tension, with these comes (4) blood poisoning from imperfect eliminations of waste stuff by the kidneys and other eminatories."

Prognosis he considers bad, on the whole; still, by judicious treatment, quite a number of patients can be saved.

Speaks of puerperal mania and meningeal hemorrhage as always to be feared as sequellæ. Under this heading he gives notes of two cases that came under his observation. The first case he considers of uræmic type, while the second is exaggerated nervous tension.

Treatment: He considers chloroform at the head of the list, stating that Dr. Barker claims that the mortality has been reduced 50 per cent. since its use; venesection, he claims, is justly entitled to the second place; those patients who will bear bleeding he advocates free, copious bleeding at the onset; spoke of nitrate amyl, nitro-glycerine, pilocarpine, etc., being advocated by some good authority. Diuretics: He favors acetate or citrate potash and digitalis. He also favors the giving of hydrogogue cathartics; of these he claims that croton oil is the most satisfactory. Restorative treatment consists of rest, nutritious diet mostly milk, good hygiene and sanitary surroundings and building up the system with tr. ferri. chor.

Dr. Virgil O. Hardon said: My experience with puerperal eclampsia amounts to nothing. In a tolerable large obstetric practice I have been so fortunate as to never have a case of this disease. Any views which I may entertain upon the subject are, therefore, the result either of *a priori* reasoning or of the study of what other men have written and said. I shall, consequently, leave the discussion to those members of the Society who have been less fortunate in this respect than I have been. I will say, however, that very early in my practice I adopted a rule, from which I have never departed, to examine at intervals the urine of every pregnant woman whom I had an opportunity to see before her labor commenced. Whenever I have found albuminuria I have kept the bowels well opened and have given diuretics freely. Of this latter class of remedies I have found acetate of potash the most satisfactory. It is, of course, impossible to say how many cases of puerperal eclampsia have been forestalled by this course, perhaps none. Form my observation of the practice of other men and from my own study and reflection, I long ago made up my mind that if I should meet with a case of this disease in my practice, I would rely upon three remedies, namely, immediate delivery, venesection and chloroform, using them in the order in which I have named them. If they failed, I would expect to see my patient die.

Dr. Peck reported four cases, the first two of which terminated fatally. He had recourse to bleeding and chloroform in both of the favorable cases. Considers venesection at the head of the list of remedial measures. Chloroform controlled the convulsions while it was being administered, but no permanent relief was obtained until blood was extracted.

Dr. Noble said he had not had a case in his own practice, but had seen a number of cases with other physicians. Thinks venesection, chloroform, morphine and bromides indicated. Does not think cathartics should be given.

Dr. Walker reported two cases which terminated favorably. Thinks cathartics do good, as both his cases seemed much benefited thereby.

Dr. Duncan had had quite a number of cases in his practice. Thinks venesection indicated in most cases. Chloroform was only beneficial while being administered. Considers morphine a valuable adjunct.

Dr. Scott believes he has succeeded in warding off two impending attacks of puerperal eclampsia by hypodermic injections of morphine.

Dr. Crow, in conclusion, said blindness is nearly always a prodrome of eclampsia. Thinks morphine contra-indicated in uræmic cases, while it is a valuable agent in those of nervous origin. Believes hysteria is frequently mistaken for eclampsia.

---

HARD CHANCRE OF THE VAGINA.—An exquisite case of hard chancre of the vagina is related by Dr. Bockhart, in the *Monath. für pract. Derm.*, No. 12, which is interesting from the manner in which it originated, as well as from the fact that the hard sore is very rarely found on the mucous membrane of the vagina, partly because of its histological formation and because, too, sores here heal quite rapidly. A woman who had never before contracted syphilis, although she had often had coitus with an infected man, experienced pain after each connection during the fortnight before she was seen, and after each intercourse drops of blood came from the vagina. Examination showed an undoubted hard chancre in the middle of the posterior vaginal wall. There were no secondary symptoms. The man had moist patches about the frænulum. The origin of the infection was this: The man, who was in the habit of performing the act several times during the night, always used at the first onset a so-called *stimulating condom* to increase the woman's genital excitation, and left it off during subsequent copulation. The instrument mentioned consisted in a thick rubber condom having rows of rubber prongs on its surface so arranged that they flatten out as the penis enters the vagina, but upon withdrawal stand out and irritate the vagina, and, undoubtedly, when often used, cause erosions. An erosion having been produced in this way, the syphilitic virus found entrance from the man's mucous patches, and the perfect induration resulted, where, without the loss of substance, spontaneous healing would have been looked for. Symptoms of constitutional syphilis soon appeared.—*Journal Cutaneous and Venereal Diseases.*

